# Supplementary figures and images for: Preoperative antihypertensives and hypotension during bladder tumor resection with oral 5-aminolevulinic acid administration
Source: PLoS One. 2025 Feb 24;20(2):e0319413. doi: 10.1371/journal.pone.0319413 (PMC11849823; doi:10.1371/journal.pone.0319413)

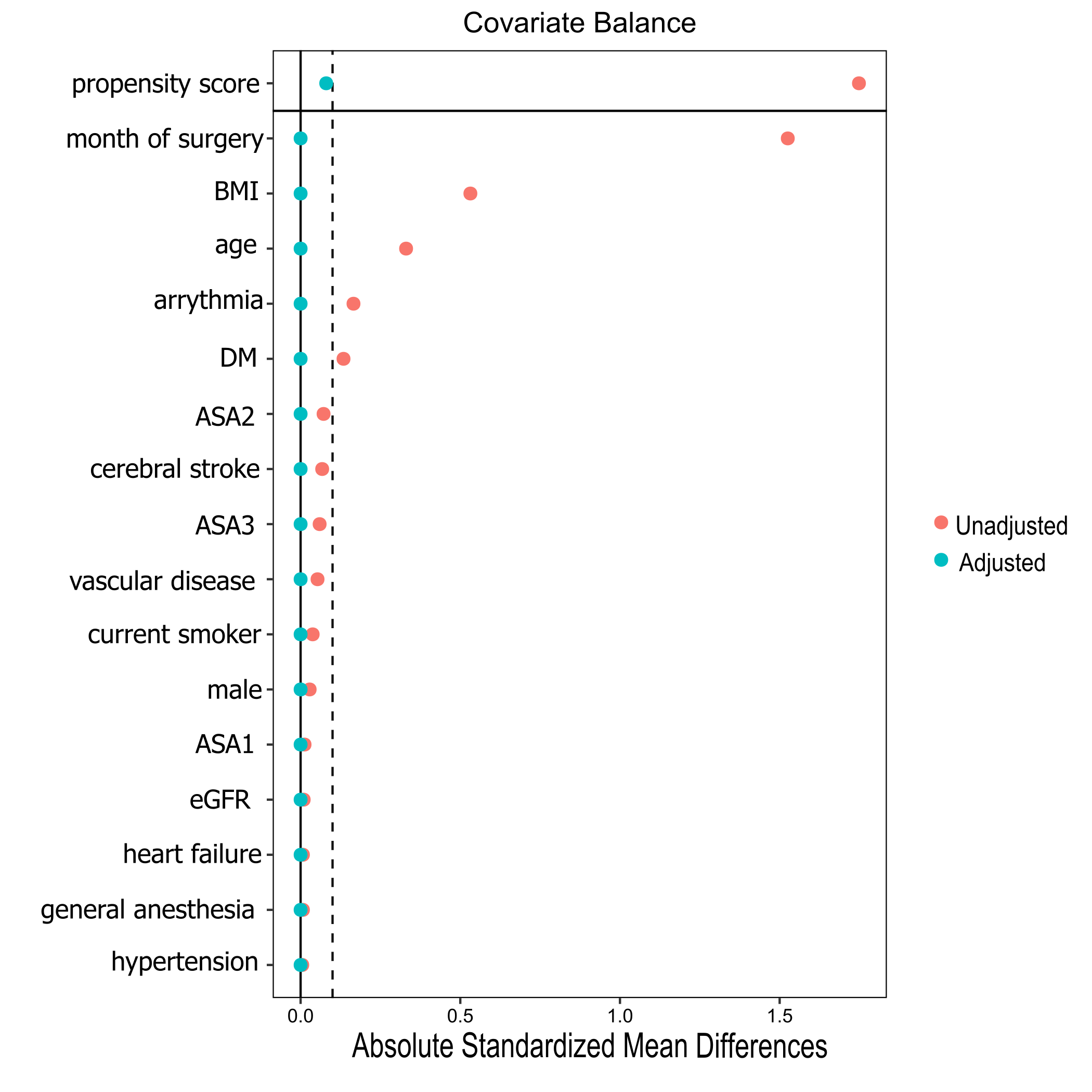

Supplement: S1 Fig — (TIF) [file pone.0319413.s001.tif]
