# Supplementary material for: Preoperative antihypertensives and hypotension during bladder tumor resection with oral 5-aminolevulinic acid administration
Source: PLoS One. 2025 Feb 24;20(2):e0319413. doi: 10.1371/journal.pone.0319413 (PMC11849823; doi:10.1371/journal.pone.0319413)
Supplement: S1 Table — (DOCX) [file pone.0319413.s002.docx]

**S1 Table. Incidence of intraoperative hypotension of average treatment effect in the overlap population model.**

|  | Discontinued group (%) | Continued group (%) | Risk ratio (95% confidence interval) | p value |
| --- | --- | --- | --- | --- |
| Adjusted model (ATO) | 50.6 | 60.0 | 1.19 (0.77–1.82) | 0.433 |

ATO, average treatment effect for the overlap population
