# Supplementary material for: Preoperative antihypertensives and hypotension during bladder tumor resection with oral 5-aminolevulinic acid administration
Source: PLoS One. 2025 Feb 24;20(2):e0319413. doi: 10.1371/journal.pone.0319413 (PMC11849823; doi:10.1371/journal.pone.0319413)
Supplement: Attachment — Submitted filename: Response_to_Reviewers_20241214.docx [file pone.0319413.s005.docx]

Our response to the comments on the manuscript: “Preoperative antihypertensives and hypotension during bladder tumor resection with oral 5-aminolevulinic acid administration” (Manuscript ID: PONE-D-24-24249R1) is listed below.

We have carefully considered all comments and made the necessary revisions to address them. Our responses to each comment are detailed below. In this document, the editor and Reviewers' comments are presented in black font, whereas our responses are in red font. In the revised manuscript, all changes are indicated in red font. A point-by-point response to all comments and suggestions is provided below:

**Journal Requirements:**

Please review your reference list to ensure that it is complete and correct. If you have cited papers that have been retracted, please include the rationale for doing so in the manuscript text, or remove these references and replace them with relevant current references. Any changes to the reference list should be mentioned in the rebuttal letter that accompanies your revised manuscript. If you need to cite a retracted article, indicate the article’s retracted status in the References list and also include a citation and full reference for the retraction notice.

Response:

We thank you for the suggestion. Accordingly, we confirmed that none of the cited papers have been retracted. Furthermore, we have ensured that all references are relevant to the context of our study and appropriately cited.

**Additional Editor Comments:**

1.　The manuscript reports the crude incidence of hypotension and adjusted results but does not elaborate on the clinical implications of these differences. A clearer discussion on why the crude incidence differs significantly while the adjusted incidence does not would strengthen the interpretation.

Response:

Thank you for your comment. The differences between the crude and adjusted incidences of hypotension reflect the impact of confounding factors. Adjusting for these confounders could allow for a more accurate assessment of the independent association between the antihypertensive drugs and intraoperative hypotension. We have added this explanation to Page 33, Lines 355–359 in the revised manuscript.

2. The secondary outcomes, such as postoperative adverse events, are briefly mentioned but not explored in detail. Briefly expanding this discussion could provide additional insights into the clinical implications of the findings.

Response:

Thank you for your valuable comment. In response, we have expanded the discussion about postoperative adverse events to provide additional insights into their clinical implications. Page 31, Lines 318–329.

3. Clarify the timeline of antihypertensive discontinuation in more detail, particularly for long-acting drugs, to better contextualize the results.

Response:

Thank you for your valuable comment. We have revised the manuscript (Page 29, Lines 282–293). In our study, antihypertensive medications were uniformly discontinued on the morning of surgery for its feasibility and ease of implementation by clinical anesthesiologists. Therefore, the exact withdrawal duration varied among individuals depending on their usual dosing schedule and time of surgery initiation. This study specifically investigated the impact of the approach of discontinuing antihypertensive medications on the morning of surgery on the occurrence of hypotension.

4. Please highlight practical recommendations, such as the need for prophylactic vasopressors, as actionable conclusions for clinicians.

Response:

Thank you for your valuable comment. We have included additional details on the need for preparing vasopressors as actionable conclusions for clinicians (Page 31, Lines 311–317).

5. The discussion could be slightly improved in a language context. Please find below some suggestions in order to improve clarity in parts of your Discussion section:

“In this study, no significant association was found between the continuation or discontinuation of antihypertensive medications on the morning of surgery and the incidence of intraoperative hypotension in adult patients undergoing scheduled TURBT after 5-ALA administration. Additionally, the incidence of adverse events during hospitalization did not differ significantly between the two groups. These findings suggest that there is no substantial justification for routinely discontinuing antihypertensive medications on the morning of surgery.”

“One possible explanation for this result is the residual effect of long-acting antihypertensive drugs. Regular intake of antihypertensives with long half-lives, even when discontinued on the morning of surgery, may result in persistent pharmacologic effects during the perioperative period. Previous studies have identified long-acting ACE inhibitors (ACE-Is) and angiotensin receptor blockers (ARBs) as risk factors for hypotension, even after a 24-hour withdrawal period [26,27]. For example, severe hypotension has been reported in patients taking amlodipine, a calcium channel blocker with a half-life of approximately 40 hours, despite discontinuation on the morning of surgery [27]. In our study, more than half of the patients were on amlodipine or combination drugs containing amlodipine, while a smaller proportion were taking telmisartan, an ARB with a long half-life. For patients regularly using long-acting antihypertensives, a withdrawal period of 12 to 24 hours may be insufficient to eliminate their effects on intraoperative blood pressure. Future research should evaluate the impact of drug type, half-life, and timing of withdrawal on the risk of hypotension.”

“Another potential factor is the potent hypotensive effect of 5-ALA itself, which may override the influence of preoperative antihypertensive medication. While our study was not designed to evaluate this interaction specifically, future research should explore this mechanism further. The pathogenesis of hypotension following 5-ALA administration remains unclear, but animal studies suggest that its metabolite, protoporphyrin IX (Pp IX), activates soluble guanylate cyclase, leading to vasodilation of systemic and pulmonary vessels. Additionally, increased vascular permeability after 5-ALA administration has been reported. Blood concentrations of Pp IX peak approximately 4–6 hours after oral administration, coinciding with the timing of surgery, which may explain the pronounced vasodilation during this period. The vasodilatory effects of anesthetic drugs likely exacerbate this phenomenon. Our findings indicate that withdrawing antihypertensive medications on the morning of surgery does not effectively prevent intraoperative hypotension. Instead, clinical strategies such as optimizing the timing of antihypertensive withdrawal or prophylactic vasopressor administration should be explored.”

“This study has several strengths. First, it examined the association between antihypertensive use on the day of surgery and intraoperative hypotension while adjusting for confounding factors. Second, the definition of hypotension used in this study was clinically meaningful and aligned with established criteria for organ perfusion and prognosis.”

“However, the study also has limitations. Imbalances in variables such as eGFR and stroke rates persisted after weighting, although these imbalances are unlikely to have overturned the results. The association between antihypertensive use and hypotension may differ depending on the type and half-life of the medications, but the study's sample size was insufficient for subgroup analyses. Additionally, the lack of information on the timing of the last antihypertensive dose precluded a detailed investigation of drug pharmacokinetics. Selection bias may have occurred, as the reasons for continuing or discontinuing antihypertensives were not documented in all cases. Finally, the absence of a standardized protocol for blood pressure management and vasopressor use during surgery introduces variability in the outcomes. Moreover, the generalizability of our findings is limited because most patients underwent general anesthesia, whereas TURBT is often performed under spinal anesthesia in other settings.”

“Conclusion

This study found no significant association between the use of antihypertensive medications on the morning of surgery and the incidence of intraoperative hypotension. Consequently, there is no substantial justification for the routine discontinuation of antihypertensive medications on the day of surgery. Further research should consider drug type, half-life, and withdrawal timing to refine preoperative management strategies.”

Response:

Thank you for your insightful suggestions. We have revised the manuscript accordingly, incorporating your recommendations to improve the clarity and readability.
