# Supplementary material for: Preoperative antihypertensives and hypotension during bladder tumor resection with oral 5-aminolevulinic acid administration
Source: PLoS One. 2025 Feb 24;20(2):e0319413. doi: 10.1371/journal.pone.0319413 (PMC11849823; doi:10.1371/journal.pone.0319413)
Supplement: Attachment — Submitted filename: Response to reviewers_20241017_tm_1104_ykplain.docx [file pone.0319413.s004.docx]

**Response to Reviewers’ Comments on the Manuscript: “Preoperative antihypertensives and hypotension during bladder tumor resection with oral 5-aminolevulinic acid administration” (Manuscript ID: PONE-D-24-24249)**

We have carefully considered all comments and made appropriate revisions to address them. Our responses to each comment are detailed below. In this text, the editor’s and reviewers' comments are presented in black font, while our responses are in red font. In the revised manuscript, revisions are indicated in red font.

Our point-by-point response to all comments and suggestions is listed below:

**Journal requirements:**

1. When submitting your revision, we need you to address these additional requirements.

Please ensure that your manuscript meets PLOS ONE's style requirements, including those for file naming. The PLOS ONE style templates can be found at

https://journals.plos.org/plosone/s/file?id=wjVg/PLOSOne_formatting_sample_main_body.pdf and

https://journals.plos.org/plosone/s/file?id=ba62/PLOSOne_formatting_sample_title_authors_affiliations.pdf.

Response: Thank you for providing these additional requirements for revising our manuscript. We have carefully reviewed the *PLOS ONE* style requirements and made the necessary adjustments to ensure compliance.

2. We note that you have indicated that there are restrictions to data sharing for this study. For studies involving human research participant data or other sensitive data, we encourage authors to share de-identified or anonymized data. However, when data cannot be publicly shared for ethical reasons, we allow authors to make their data sets available upon request. For information on unacceptable data access restrictions, please see http://journals.plos.org/plosone/s/data-availability#loc-unacceptable-data-access-restrictions.

Before we proceed with your manuscript, please address the following prompts:

a) If there are ethical or legal restrictions on sharing a de-identified data set, please explain them in detail (e.g., data contain potentially identifying or sensitive patient information, data are owned by a third-party organization, etc.) and who has imposed them (e.g., a Research Ethics Committee or Institutional Review Board, etc.). Please also provide contact information for a data access committee, ethics committee, or other institutional body to which data requests may be sent.

b) If there are no restrictions, please upload the minimal anonymized data set necessary to replicate your study findings to a stable, public repository and provide us with the relevant URLs, DOIs, or accession numbers. Please see http://www.bmj.com/content/340/bmj.c181.long for guidelines on how to de-identify and prepare clinical data for publication. For a list of recommended repositories, please see https://journals.plos.org/plosone/s/recommended-repositories. You also have the option of uploading the data as Supporting Information files, but we would recommend depositing data directly to a data repository if possible.

Please update your Data Availability statement in the submission form accordingly.

Response:

Thank you for your guidance regarding data sharing. We fully understand the importance for making data available to ensure transparency and reproducibility in scientific research. There are ethical restrictions which prevent the public sharing of minimal data for this study because the data contain sensitive participant information. Data are available upon request from Yokohama Rosai Hospital's Research Ethics Committee via email (rinri@yokohamah.johas.go.jp) for researchers who meet the criteria for access to confidential data.

Reviewer #1:

This is a fascinating study that examines the pros and cons of antihypertensive drug withdrawal in photodynamic diagnostic TURBT with oral 5-ALA. However, I think that the number of patients is too small to prove the superiority and non-inferiority of the intervention of discontinuing antihypertensives, as the authors state in their limitation. Also, as the authors state, the half-life of the antihypertensive drugs was not taken into account, and simply stopping them on the day of surgery may not have made a difference. From the above, the results of this study provide some insight, but unfortunately, I do not think we can make the judgment that "withdrawal of antihypertensive drugs on the day of surgery is meaningless". Further additional study on this topic is warranted.

Response:

We totally agree with you. We have already mentioned about this in the limitations section. We agree that further research is warranted. Furthermore, we are committed to continuing our efforts to address this important issue.

Regarding patient background

Around 2019 (when the paper Nohara T et al. Int J Urol 26(11), 1064-1068, 2019 was published), intraoperative hypotension due to oral 5-ALA began to attract attention. After March 2020, there seems to be a sharp increase in cases of antihypertensive drug withdrawal on the day of surgery. Is this a departmental policy or an individual decision? We would like to know the reason for this.

Response:

This is a departmental policy. Due to concerns about perioperative hypotension, antihypertensive medications were discontinued on the day of surgery from March 2020, following discussions between the director of the department of anesthesiology and urologists (as noted Page 6 to 7, Lines 84 to 88). However, several patients did not adhere to this policy probably due to delays in communicating the policy change within the department, errors in entering preoperative instructions, and miscommunication in the wards. The exact reasons for non-adherence could not be determined even after reviewing the medical records (as noted Page 14, Lines 211 to 214), and this has been added as a limitation to our study (Page 33, Lines 353 to 357).

Definition of hypotension

The primary outcome measure was the incidence of intraoperative hypotension. Hypotension was defined as MBP of less than 65 mmHg for ≥20 min, Although this definition was meaningful, the explanation of the definition was not well described. For example, if MBP ≥ 65 mmHg is reached even once during MBP< 65 mmHg for 20 min, due to bolus administration of vasoactive agents or fluid infusion, which would you consider there is a hypotensive event or not? Furthermore, what about intervals between blood pressure measurements for 20 minutes? The definition should be described in more detail.

Response:

We define hypotension as an MBP < 65 mmHg that persisted for more than 20 minutes.

Therefore, as in the scenario you describe a brief increase in MBP due to a bolus administration of vasoactive agents or fluid infusion could break up the hypotensive duration to less than 20 minutes, which would not be considered a hypotensive event. The interval between blood pressure measurements was typically 2.5–5 min. We have added this clarification to the revised manuscript (Page 8 Line 114 and Page 14, Lines 214 to 215).

Regarding RAS inhibitor

A previous study showed that regular ARB use was a risk for perioperative hypotension (defined as SBP < 80 mmHg at induction of anesthesia with hypotension) even if the ARB was withdrawn on the day of surgery (Nohara T et al. Int J Urol 26(11), 1064-1068, 2019). Is it possible that hypotensive events occur more frequently in patients who regularly use RAS inhibitors?

Response:

Thank you for your insightful comment. As noted in previous literature, patients who regularly use RAS inhibitors may be at a higher risk of experiencing hypotensive events. Our study was designed to evaluate the impact of continuing or discontinuing antihypertensive medications on the morning of surgery on intraoperative hypotension. In our institution, discontinuing RAS inhibitors on the day of surgery is a standardized practice. Therefore, patients who were on regular use of RAS inhibitors as a single agent were excluded from this study. This exclusion may have contributed to finding a lower overall incidence of hypotension compared with previous reports.

For reference, we calculated the incidence of hypotension in patients who were included in the study despite using RAS inhibitors regularly due to the concurrent use of other regular antihypertensive medications, such as calcium channel blockers. Among these cases, 32 out of 51 cases (62.7%) experienced hypotension. Specifically, in the discontinued group, 16 out of 31 cases (51.6%) experienced hypotension, while in the continued group, 16 out of 20 cases (80.0%) experienced hypotension. However, our study was not planned to compare patients who regularly used RAS inhibitors with those who did not. Therefore, we did not analyze this specific aspect.

Regarding anesthesia.

Why do almost all patients at your institution receive general anesthesia? Is there any reason?

Response:

We tend to select general anesthesia for several reasons.

The obturator nerve block procedure is time consuming. In certain cases, wherein an obturator nerve block or muscle relaxation is required, we tend to choose general anesthesia. Additionally, the possibility of prolongation of surgery also contributes to making this decision.

Conclusion

The author concluded that no substantial justification was provided for routinely discontinuing antihypertensive medications. I would like to know what the authors consider from this result. For example, “antihypertensives should be withdrawn on the day of surgery,” “it should be continued,” “whether to continue or discontinue antihypertensive medication should be considered in each individual case,” or “further analyses are warranted.“ please consider describing your opinion.

Response：

We suggest that further study is necessary, particularly studies that take into account the type of antihypertensive medication, its half-life, and the duration of its discontinuation. We have added this consideration to the Conclusion section (Page 34, Lines 370 to 371).

Reviewer #2

This is an intriguing study that evaluated the relationship between preoperative antihypertensive medication and hypotension during PDD-TURBT with oral 5-ALA administration. The assessment was conducted by adjusting for background factors using the overlap weighting method. The authors presented results indicating that, after adjusting for background factors, the presence or absence of preoperative antihypertensive medication on the day of surgery does not affect the incidence of intraoperative hypotension. This finding is considered to be highly beneficial in clinical practice.

Response：Thank you for recognizing the clinical value of our study.

Please address the following points:

1. The use of both the ATT and ATO models might be causing confusion. It would be better to use only one of these models.

Response：Thank you for your advice. To ensure the robustness of our results, we used both models in our analysis. Since the main analysis was adjusted using the ATT model, we have moved the results of the ATO model to the supporting information.

2. Tables 1, 3, and 4: Please present the percentages for the Discontinued group and the Continued group in the adjusted model.

Response：

We have revised Tables 1, 3, and 4.

3. Discussion: The authors suggest that one reason for the lack of a significant association between the continuation or discontinuation of antihypertensive drugs on the morning of surgery and the incidence of intraoperative hypotension is the residual effects of the antihypertensive drugs, which may be correct. However, it is also possible that the hypotensive effect of 5-ALA is so strong that it overrides whether or not antihypertensive drugs are taken preoperatively. Previous studies have shown mixed results, with some indicating that the use of antihypertensive drugs is related to the development of hypotension and others indicating no such relationship. Ideally, this study might have been better if it had included a comparison among three groups, including a group of patients who were not taking antihypertensive medications. At the very least, the authors need to address this issue in the Discussion section.

Response:

Thank you for your insightful comments. We agree with you that the hypotensive effect of 5-ALA might have been so strong that it overrode the impact of whether or not antihypertensive drugs were taken preoperatively. As you correctly pointed out, clarifying this would require a comparison between the three groups, including a group of patients who did not regularly take antihypertensive medications. However, our study was specifically designed to address a clinical question: whether antihypertensive medications should be continued on the morning of surgery in patients undergoing TURBT after taking 5-ALA. It was not designed to explore the mechanisms behind the effects of antihypertensive drug discontinuation on intraoperative hypotension. Therefore, determining the underlying mechanism is beyond the scope of this study. We appreciate your suggestion and consider this an important area for future research. We have incorporated this point into the revised manuscript (Page 29, Lines 290 to 293).

Reviewer #3:

This study examined the effect of antihypertensive medications on hypotension in patients taking ALA before surgery, defining hypotension as a mean blood pressure (MBP) of less than 65 mmHg for 20 minutes or longer. The authors conclude that the adjusted results suggest no significant association between the continuation of antihypertensive medications and the incidence of intraoperative hypotension to justify routine discontinuation of antihypertensive medications, but this alone does not seem to be novel. Therefore, please consider the following points.

Major points

• Although you report no significant association between the continuation of antihypertensive medication and the incidence of intraoperative hypotension, was there an association between the continuation of antihypertensive medication and the response to boosting medication after the occurrence of hypotension? Please add that point to the manuscript and add a new table if necessary.

Response:

Thank you for your insightful comments. We recognize the importance of your suggestion. However, there are several challenges to accurately measure response to boosting medication after the occurrence of hypotension in this observational study. First, defining the time when an increase in blood pressure could be said to be a response to boosting medication is difficult. In addition, intraoperative blood pressure could be influenced by various factors, including surgical procedures, which could occur close to the time of boosting medication administration. Therefore, making isolating the effect of the boosting medication difficult. Second, in clinical practice, vasopressors are not always administered only after hypotension occurrence. They are sometimes given prophylactically based on the trend in blood pressure. Determining whether the administration of vasopressors could be prophylactic or therapeutic based on anesthesia records is difficult. Additionally, our study defines hypotension as a MBP of less than 65 mmHg persisting for ≥ 20 minutes. Depending on the anesthesiologist's judgment and patient’s usual blood pressure, it is likely that vasopressors were often administered to treat or prevent hypotension when MBP fell below 65 mmHg or was about to do so. The fact that hypotension persisted for ≥ 20 minutes despite these treatments may partially reflect a low responsiveness to boosting medication. In our study, intraoperative blood pressure management was left to the discretion of the attending anesthesiologist; there was no standardized policy regarding blood pressure targets and the decision to use vasopressors. This is one of the common limitations of retrospective observational studies. We have added this point to the Discussion section (Page 33, Lines 357 to 362). We thank you for the questions you have raised that are indeed important. We suggest that well-designed prospective studies are needed to address this question.

Minor points.

• There have been several reports of ALA-induced hypotension from urology and anesthesiology departments, with varying definitions of hypotension. Why is hypotension defined as a mean blood pressure (MBP) of less than 65 mmHg for more than 20 minutes? Please include a brief explanation of why in your manuscript.

Response:

We appreciate your suggestion. We have already provided a brief explanation in the revised manuscript on page 8, lines 113 to 118. We hope that this could addresses your comment; we are open to further discussion if needed.

Reviewer #4: The authors analysed the frequency of hypotension in the continuation and discontinuation of antihypertensive medication before TURBT. This paper is considered valuable in demonstrating that continuation of antihypertensive medication is feasible. My opinion is as follows.

Major.

What is the time between antihypertensive drug administration and TURBT? Is there an association between that time and hypotension? Is it conceivable, for example, that hypotension is less likely to occur if antihypertensive drugs are taken only in the morning and surgery is performed in the afternoon?

Response:

The exact time of the last antihypertensive drug administration is not usually documented in medical records. Therefore, we could not evaluate the association between the time from antihypertensive drug administration to TURBT and hypotension accurately. This limitation of our study has already been mentioned on lines 350 to 353 of page 32 to 33. However, as you pointed out, the association between the washout period of antihypertensive drugs and hypotension is an important issue. Future studies should clarify the actual last time antihypertensive medication was taken and take into account the washout period.

Is the definition of hypotension appropriate? Can hypotension with various risks be left untreated for 20 minutes?

Response:

Our definition of hypotension was based on several previous studies indicating that intraoperative MBP below 60–70 mmHg was associated with myocardial infarction, acute kidney injury (AKI), and death (reference number 17 in the manuscript) and 20 min or longer duration increased the risk of AKI and myocardial infarction (reference number 18 in the manuscript). In our study, intraoperative blood pressure management was left to the discretion of the attending anesthesiologist. Moreover, there was no standardized policy regarding blood pressure targets and decision to use vasopressors. This limits the interpretation of results. This is one of the common limitations of retrospective observational studies. We added this meaning to the Discussion section (Page 33, Lines 357 to 362). Depending on the anesthesiologist's judgment and patient’s usual blood pressure, administering vasopressors to treat hypotension when MBP falls below 65 mmHg is a common practice, rather than leave it. In our data, vasopressors were used in 87.3% of patients with hypotension, meaning they persisted hypotension despite the use of vasopressors. In other words, the fact that MBP <65 mmHg persisted for ≥ 20 minutes may partially indicate that the patient was less likely to respond to vasopressors. The fact that approximately half of the patients were classified as hypotensive was a point that we considered. Strategies are needed for the effective treatment of hypotensive in patients after 5-ALA administration who are unlikely to respond to ephedrine or phenylephrine.

It is stated that the assessment of preoperative blood pressure in the 2ndary Outcome was not performed due to many missing values, but if it is a retrospective observational study, there is no need to include it in the methods section in the first place.

Response:

Thank you for your comment. Accordingly, we have removed the relevant text from the Methods section.

Minor.

What is the R ver?

Response:

We used the R version 4.2.0. We added this information to Page13, Line 195.

Figure 1 - Not appropriate as there are two similar statements in the same block.

Response:

Thank you for pointing this out. Accordingly, we have corrected Fig 1.

If the two groups are adjusted for patient background, consider presenting pre- and post-adjustment data.

Response:

We have revised Tables 1, 3, and 4.

There is no mention of hepatotoxicity, the most likely adverse effect of ALA.

Response:

We recognize that one of the side effects of 5-ALA is hepatotoxicity, as you have pointed out, but this study was primarily on the incidence of intraoperative hypotension. Therefore, we did not mention hepatotoxicity.

Reviewer #5:

Thank you very much for giving me the opportunity to review this manuscript. The study clarified the effect of antihypertensive drugs on ALA-related hypotension during surgery. The manuscript is very well written and scientifically solid, but I have raised some questions that should be addressed.
Response:

Thank you for your positive comments.

Major:
1. Line 96
The definition of hypotension (MBP less than 65 mmHg for > 20 min) is a quite severe situation. How did you manage the hypotension during surgery? According to the Table 3, about 60% of patients were administered with vasoactive agents including ephedrine. Do you mean MBP were less than 65 mmHg even in those patients? If so, the vasoactive agents seem not working for those patients.
As the management of hypotensive patients after PDD-ALA is important for the readers, please describe how the BP fluctuated during surgery.

Response:

In our study, intraoperative blood pressure management was left to the discretion of the attending anesthesiologist; there was no standardized policy regarding blood pressure targets and the decision to use or dose of vasopressors. This limits the interpretation of results. This is one of the common limitations of retrospective observational studies. We added this meaning to the Discussion section (Page 33, Lines 357 to 362). Of the cases that presented with hypotension, most of cases used some type of vasopressor during surgery. The dose of ephedrine and phenylephrine tended to be higher in patients who presented with hypotension, which might indicate that ephedrine or phenylephrine was not fully effective despite repeated administration (Page 31, Lines 325 to 330).

As you mentioned, intraoperative blood pressure is an important information. We have provided intraoperative blood pressure data in Fig 3.

Minor:
1. Line 215
91.4+17.6=109. Please correct the number.

Response:

Thank you for pointing this out. We have corrected the manuscript (Page 14, Lines 209 to 211).

Reviewer #6:

This manuscript studied the effect of continuing antihypertensives on intraoperative hypotension in patients who underwent TURBT with 5-ALA administration. Hypotension is one of major concerns related to 5-ALA administration. In this study, the adjusted results suggested no significant association between the continuation of antihypertensive medication and the incidence of intraoperative hypotension. The manuscript was well written and revealed attractive results for urologists and anesthesiologists in the clinical practice.
I have several questions and comments to this manuscript as follows:
Response:

Thank you for your positive comments.

Major
1. This retrospective study included a total of 132 cases (108 patients) for the analysis. It seemed to be relatively small to make a conclusion. The authors may want to increase the number of patients or perform an external validation.

Response:

We acknowledge that the sample size of this study could be relatively small. Therefore, strong conclusions based this study alone might not be made. We suggest that our study should serve as a priming ground for the publication of additional evidence from other institutions on this topic. We consider that our study would be one of the pieces of evidence for future conclusions on this topic.

2. Based on the results of this study, the authors should show the clinical implication. Should we stop or continue the antihypertensives on the morning of surgery?

Response:

As noted above, strong conclusions could not be drawn based on the findings in this study alone. Future studies are needed.

3. The authors concluded that “no substantial justification was provided for routinely discontinuing antihypertensive medications.” As the authors discussed in the manuscript, discontinuation on the morning of surgery may not be enough to eliminate the effects of antihypertensive drugs. Because the discontinuation period might affect the intraoperative hypotension, the authors should modify the conclusions as limited to the drug withdrawal on the morning of surgery.
Response:

Thank you for pointing this out. We have revised the manuscript (Page 34, Lines 369 to 370).

Minor
1. Please include the number of regular used antihypertensives for each group in table 1.

Response:

We added the number of regular used antihypertensives in each group to Table 1.

2. Please indicate the type of continued antihypertensives for the continued group in table 1.

Response:

We added the type of continued antihypertensives in the continued group to Table 1.
