# Supplementary material for: Preoperative antihypertensives and hypotension during bladder tumor resection with oral 5-aminolevulinic acid administration
Source: PLoS One. 2025 Feb 24;20(2):e0319413. doi: 10.1371/journal.pone.0319413 (PMC11849823; doi:10.1371/journal.pone.0319413)
Supplement: Attachment — Submitted filename: Response_to_Reviewers_20250125.docx [file pone.0319413.s006.docx]

Response to Reviewers’ Comments on the Manuscript: “Preoperative antihypertensives and hypotension during bladder tumor resection with oral 5-aminolevulinic acid administration” (Manuscript ID: PONE-D-24-24249R2).

We have carefully considered all comments and made the necessary revisions to address them. Our responses to each comment are detailed below. In this document, the editor’s and reviewers' comments are presented in black font, whereas our responses are in red font. In the revised manuscript, all changes are indicated in red font. A point-by-point response to all comments and suggestions is provided below:

**Journal Requirements:**

Please review your reference list to ensure that it is complete and correct. If you have cited papers that have been retracted, please include the rationale for doing so in the manuscript text, or remove these references and replace them with relevant current references. Any changes to the reference list should be mentioned in the rebuttal letter that accompanies your revised manuscript. If you need to cite a retracted article, indicate the article’s retracted status in the References list and also include a citation and full reference for the retraction notice.

Response:

We confirm that none of the cited papers have been retracted. Furthermore, we have ensured that all references are relevant to the context of our study and appropriately cited.

**Additional Editor Comments:**

1. Table 1 content overload: Table 1 contains an overwhelming amount of demographic and clinical information. While comprehensive, it hinders readability and distracts from the key findings. Please restrict Table 1 to the most relevant demographic and clinical variables (e.g., age, sex, BMI, ASA-PS, type of antihypertensives, etc). Move less critical variables to a supplementary table.

Response:

Thank you for your comment. We have revised Table 1 to focus on the most relevant variables.

2. Table 3 should include only vasoactive drugs that were actually used in the studied population. Please correct and provide adequate legends for this table.

Response:

Thank you for your valuable comment. We have revised Table 3. Additionally, we have provided appropriate legends for the table.

3. The discussion is comprehensive but can be better organized. Consider grouping similar ideas together for improved readability. Suggestions for specific corrections are provided below:

- Line 265-266: "No significant association was found between continuing or discontinuing antihypertensive medications on the morning of surgery and intraoperative hypotension during scheduled TURBT following 5-ALA administration."

Response:

Thank you for your suggestions. We have revised the manuscript accordingly, incorporating your recommendations to improve the clarity and readability.

- The phrase "persistent pharmacologic effects during the perioperative period" is unclear without elaboration. Specify the clinical relevance of these persistent effects. For example: "...result in persistent pharmacologic effects during the perioperative period, such as attenuation of blood pressure fluctuations."

Response:

Thank you for your suggestions. We have revised the manuscript accordingly, incorporating your recommendations to improve the clarity and readability.

- Line 286-289: The description of withdrawal periods is informative but repetitive. Condense the explanation: "Withdrawal periods varied between patients, with some experiencing approximately 28 hours and others about 12 hours, depending on their usual medication schedule."

Response:

Thank you for your suggestions. We have revised the manuscript accordingly, incorporating your recommendations to improve the clarity and readability.

- Line 296-302: Lack of clarity regarding the hypothesis of 5-ALA effects. Explicitly state how this impacts clinical decisions: "These findings suggest that the vasodilatory effects of 5-ALA, compounded by anesthetic drugs, may outweigh the influence of preoperative antihypertensive withdrawal. This highlights the need for tailored strategies, such as timing adjustments or pharmacologic interventions."

Response:

Thank you for your suggestions. We have revised the manuscript accordingly, incorporating your recommendations to improve the clarity and readability.

- Line 321-329: Blood pressure monitoring is discussed but lacks actionable clinical recommendations. Include specific strategies: "To mitigate these risks, we recommend frequent blood pressure monitoring and considering preoperative fluid loading in patients administered 5-ALA."

Response:

Thank you for your suggestions. We have revised the manuscript accordingly, incorporating your recommendations to improve the clarity and readability.

- Line 331-339: The comparison to the phase III study could emphasize its implications. Provide a clearer explanation: "Differences in patient demographics and comorbidities likely contributed to the higher incidence of hypotension in this study. This emphasizes the importance of patient selection and stratification in future trials."

Response:

Thank you for your suggestions. We have revised the manuscript accordingly, incorporating your recommendations to improve the clarity and readability.

- Line 343-350: The efficacy of vasopressors is discussed but not thoroughly analyzed. Elaborate on why ephedrine and phenylephrine might be less effective and propose alternatives: "The limited efficacy of ephedrine and phenylephrine suggests the need for alternative strategies, such as using noradrenaline as a first-line vasopressor in patients receiving 5-ALA."

Response:

Thank you for your suggestions. We have revised the manuscript accordingly, incorporating your recommendations to improve the clarity and readability.

- Line 354-359: The statement about confounding factors is strong but lacks context. Include a specific example of a confounding factor: "For instance, patients with advanced age or cardiovascular disease may require individualized adjustments, as these factors significantly influence blood pressure dynamics."

Response:

Thank you for your suggestions. We have revised the manuscript accordingly, incorporating your recommendations to improve the clarity and readability.

- Line 363-371: The limitations section is detailed but can be more concise. Combine related points: "This study is limited by its retrospective design, imbalances in variables after weighting, and insufficient sample size for subgroup analyses. Furthermore, the lack of standardized protocols for blood pressure management may have introduced variability in outcomes."

Response:

Thank you for your suggestions. We have revised the manuscript accordingly, incorporating your recommendations to improve the clarity and readability.

- Line 377-382: The conclusion repeats earlier points. Focus on actionable insights: "This study supports the continued use of antihypertensive medications on the morning of surgery and highlights the need for individualized perioperative strategies based on drug characteristics and patient profiles."

Response:

Thank you for your suggestions. We conclude that there is no evidence to support discontinuing antihypertensive medications on the morning of surgery. This does not imply a recommendation to continue their use. Therefore, based on your suggested revision, we have rewritten the following statement. “This study found no evidence to support discontinuing antihypertensive medications on the morning of surgery and underscores the need for individualized perioperative strategies based on drug characteristics and patient profiles.”
